# Normobaric hyperoxia protects the blood brain barrier through inhibiting Nox2 containing NADPH oxidase in ischemic stroke

**DOI:** 10.1186/2045-9912-1-22

**Published:** 2011-09-06

**Authors:** Wenlan Liu, Qingquan Chen, Jie Liu, Ke Jian Liu

**Affiliations:** 1 Department of Pharmaceutical Sciences, University of New Mexico Health Sciences Center, Albuquerque, NM 87131, USA; 2 Department of Neurology, University of New Mexico Health Sciences Center, Albuquerque, NM 87131, USA

## Abstract

Normobaric hyperoxia (NBO) has been shown to be neuro- and vaso-protective during ischemic stroke. However, the underlying mechanisms remain to be fully elucidated. Activation of NADPH oxidase critically contributes to ischemic brain damage via increase in ROS production. We herein tested the hypothesis that NBO protects the blood-brain barrier (BBB) via inhibiting gp91^phox ^(or called Nox2) containing NADPH oxidase in a mouse model of middle cerebral artery occlusion (MCAO). Wild-type C57/BL6 mice and gp91^phox^knockout mice were given NBO (95% O_2_) or normoxia (21% O_2_) during 90-min MCAO, followed by 22.5 hrs of reperfusion. BBB damage was quantified by measuring Evans blue extravasation. The protein levels of matrix metalloproteinase-9 (MMP-9), tight junction protein occludin and gp91^phox ^were assessed with western blot. Gel zymography was used to assess the gelatinolytic activity of MMP-9. In the wild type mice, cerebral ischemia and reperfusion led to remarkable Evans blue extravasation, significantly increased gp91^phox ^and MMP-9 levels and decreased occludin levels in the ischemic brain tissue. In gp91^phox ^knockout mice, the changes in Evans blue extravasation, MMP-9 and occludin were at much smaller magnitudes when compared to the wild type. Importantly, NBO treatment significantly reduced the changes in all measured parameters in wild type mice, while did not cause additional reductions in these changes when gp91^phox ^was knocked out. These results indicate that activation of Nox2 containing NADPH oxidase is implicated in the induction of MMP-9, loss of occludin and BBB disruption in ischemic stroke, and inhibition of Nox2 may be an important mechanism underlying NBO-afforded BBB protection.

## Background

Normobaric hyperoxia (NBO) has been shown to effectively reduce tissue infarction and protect the blood brain barrier (BBB) in animal ischemic stroke models [1-6]. These neuro- and vaso-protective effects make NBO a promising approach to expand the narrow time window of the reperfusion therapies for ischemic stroke [7]. Indeed, recent studies showed that NBO treatment during cerebral ischemia significantly reduced the neurovascular complications in delayed tPA treatment in a rat model of ischemic stroke [8,9]. In human studies, NBO treatment was associated with improvements in clinical deficit and survival in selected stroke patients [10,11]. Increasing oxygen level, particularly over-oxygenation, with oxygen therapy may result in oxidative stress and free radical damage. Interestingly, NBO treatment for ischemic stroke does not increase oxidative stress [2], instead, it may decrease reactive oxygen species (ROS) production [3]. However, it remains to be elucidated how NBO affects ROS production in the ischemic brain.

Several oxidant enzyme systems, such as xanthine oxidase, mitochondrial respiratory chain and NADPH oxidase have been identified as important source of ROS in the brain and contribute to oxidative brain injury following cerebral ischemia and reperfusion [12,13]. Accumulating evidence from animal stroke studies suggests that Nox is strongly implicated in the oxidative damage to the neuronal tissue and the BBB in ischemic stroke [14-18]. NADPH oxidase was first found in phagocytes, which is assembled from a membrane spanning flavocytochrome *b*558, composed of Nox2 (also called gp91^phox^) and p22^phox ^and four cytosolic factors (p47^phox^, p67^phox^, p40^phox^, and Rac) that associate with the flavocytochrome to form an active enzyme [19]. Recently, several novel homologs of the catalytic, electron carrier component of NADPH oxidase (gp91^phox ^or Nox2) have been described in a variety of nonphagocytic cells, including Nox1, Nox3, Nox4, Nox5, Duox1 and Duox2 [20]. Among these homologies, only Nox1, Nox2 and Nox4 are found in brain tissue, and Nox2 expression is abundant in glia cells and endothelial cells, two major cellular components of the BBB [21,22]. Deletion of Nox2 (or gp91^phox^) results in reduced BBB damage in mouse models of ischemic stroke [22-27]. Nox2-derived ROS can directly oxidize phospholipid bilayer membrane to result in membrane disruption [28]. It can also indirectly interfere with the barrier function of the BBB through ROS-mediated stimulation of VEGF, monocyte chemoattractant protein-1, and matrix metalloproteinase-9 (MMP-9) [29-32]. Our previous studies showed that NBO treatment resulted in parallel reductions in MMP-9 and gp91^phox ^expression in ischemic neuronal tissue and microvessels [3,4,16,32]. However, it remains to clarify whether there is a causal link between Nox2 containing NADPH oxidase and MMP-9 induction in the ischemic brain and whether NBO protects the BBB through acting on Nox2.

In this study, we addressed these important questions on a mouse model of middle cerebral artery occlusion (MCAO) by comparing BBB damage, MMP-9 induction and the changes in tight junction protein claudin-5 and occludin between wild-type and gp91^phox ^knockout mice. In addition, we also determined whether NBO induces any additional changes to these parameters when gp91^phox ^was genetically deleted.

## Materials and methods

### Mice model of focal cerebral ischemia

All experimental protocols were approved by the laboratory animal care and use committee of the University of New Mexico, and were performed in accordance with animal protection guidelines. Male gp91^phox ^knockout mice (Jackson Laboratories, Bar Harbor, Ma, USA) and wild-type C57/BL6 mice (Charles River Laboratories, Wilmington, Ma, USA), aged between 7-9 weeks, were anesthetized with isoflurane (4% for surgical induction, 1.5% for maintenance) during surgical procedure.

Focal cerebral ischemia was established by introducing a silicone-coated nylon monofilament into the right common carotid artery and advancing it along the internal carotid artery till establishing a proximal occlusion of the right middle cerebral artery (MCA). After 90 min occlusion, the filament was withdrawn to allow reperfusion for another 22.5 hr. Body temperature of the mice was maintained with a heating pad to keep the rectal temperature between 37°C to 38°C.

The success of the surgery was confirmed by 2% 2,3,5-triphenyltetrazolium chloride (TTC) staining of a 1-mm thick brain slice 3 mm away from the tip of the frontal lobe as we described previously [32]. All mice included in this study showed typical tissue infarction in the MCA territory of TTC-stained sections, indicating successful MCAO.

### Normobaric hyperoxia treatment

Wild-type and gp91^phox ^knockout mice were randomly assigned to normoxic and NBO group. Five min after the onset of MCAO, mice were put into separated individual air-tight boxes which were ventilated (3 L/min) with medical air (21% O_2_) or a gas mixture of 95% O_2 _+ 5% CO_2 _during the 90 min ischemia. This specific gas mixture was shown to be neuroprotective in our previous studies using a rat model of stroke [3,4,32].

### Measurement of BBB permeability

One hour before the end of the reperfusion, 2% Evans blue in normal saline (6 mL/kg body weight) was injected into the tail vein. At the end of the experiment, mice were deeply anesthetized with isoflurane and transcardially perfused with PBS till colorless fluid was obtained from the right atrium. Brains were quickly taken out and stored at -80°C till analysis. To measure the amount of Evans blue dye in the brain, tissues from left or right hemisphere were separately homogenized in 1 mL 50% trichloroacetic acid. The fluorescence intensity of each collected supernatant was measured on a microplate fluorescence reader with excitation wavelength of 600 nm and emission wavelength of 650 nm. The total Evan's blue content (ng) in each sample was calculated according to the external standard curve. The difference of dye content between ischemic and nonischemic hemispheric tissue reflected the extent of BBB damage.

### Gelatin zymography analysis of MMP-2/9

MMP-2/9 activity was analyzed by gelatin zymography as we described previously [33]. Briefly, brain tissue was homogenized with lysis buffer containing 50 mM Tris, 150 mM NaCl, 5 mM CaCl_2_, 0.05% Brij-35, 0.02% NaN_3 _and 1% Triton X-100. MMP-2/9 were extracted from tissue homogenates using gelatin sepharose beads (GE Healthcare). Samples were electrophoresed on 10% sodium dodecylsulfate-pholyacrylamide gels containing 1 mg/mL gelatin under non-reducing conditions. Gels were washed in 2.5% Triton X-100 and then incubated for 48 hrs with the developing buffer containing 50 mM Tris, 5 mM CaCl_2_, 0.2 mM NaCl and 0.02% Brij-35. After incubation, gels were stained with 0.125% Coomassie blue R-250 to visualize clear gelatinolytic bands. A mixture of human MMP-2/9 (Invitrogen) was used as standards.

### NADPH oxidase activity assay

Lucigenin-enhanced chemiluminescence method was used in measuring the enzyme activity of NADPH oxidase in tissue homogenates as we recently described [16]. In brief, 5 μM lucigenin and 100 μM NADPH (Sigma) were added to tissue extracts. Immediately after the addition of NADPH, chemiluminescence was measured with a luminometer (Model TD-20, Turner designs, Sunnyvale, CA, USA). For each sample, 30 seconds integrated luminescence was measured and repeated for 5 times. After measurement, the samples were recollected and protein quantification was calculated using Bradford reagent (Bio-rad). The activity of NADPH oxidase was calculated as the average of the 5 repeats and was expressed as relative luminescence units per minutes per mg protein.

### Western blot analysis of gp91^phox^, MMP-9, occludin and claudin-5

Brain samples were homogenized and then lysed with RIPA buffer (Santa Cruz Biotech). Protein extracts (50 μg of total protein) were boiled and electrophoresed in 10% sodium dodecyl sulfate-polyacrylamide gels, then transferred onto nitrocellulose membranes (Bio-Rad). Membranes were blocked with tris-buffered saline containing 0.1% Tween-20 (TBS-T) and 5% non-fat milk at room temperature for 1 hr prior to overnight incubation at 4°C with primary antibodies against gp91^phox ^(1:1000 dilution, BD Transduction Laboratory, Lexington, KY, USA), MMP-9 (1:500 dilution, Cell Signaling, Boston, Ma, USA), occludin or claudin-5 (both at 1:1000 dilution, Invitrogen). After washing with TBS-T, membranes were then incubated for 1 hr at room temperature with horseradish peroxidase-conjugate corresponding secondary antibodies (anti-mouse, anti-goat or anti-rabbit, Santa Cruz). The membranes were then developed with the supersignal west pico horseradish peroxidase substrate kit (Pierce, Rockford, IL, USA) and photographed on a Kodak 4000 image station (Caresteam molecular imaging). To control sample loading and protein transfer, the membrane were stripped and reprobed with β-actin antibody (1:1000 dilution, Santa Cruz).

## Results

### NBO reduces gp91^phox ^protein levels in ischemic brain tissue

**Figure 1:**
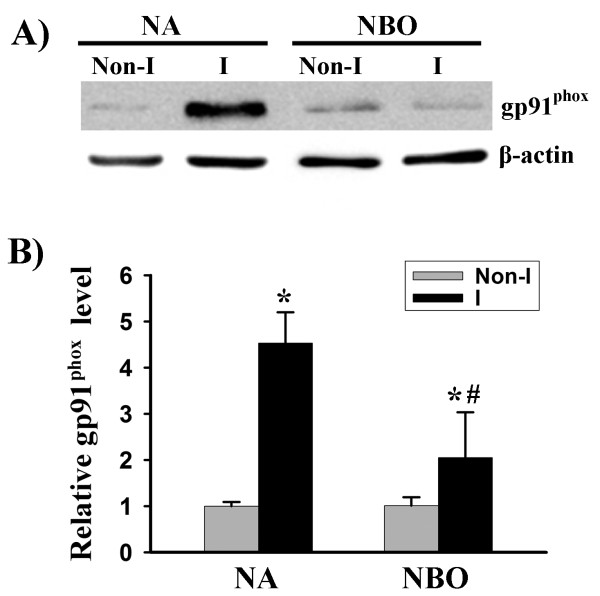
**NBO treatment inhibits gp91^phox ^induction in ischemic brain tissue after 90-min MCAO with 22.5 hrs of reperfusion**. Nonischemic (Non-I) and ischemic (I) hemispheric brain tissue was homogenized for analyzing gp91^phox ^protein levels with western blot. As a loading control, the blots were stripped and reprobed with β-actin. **A) **Representative blots of gp91^phox ^and corresponding β-actin. **B) **The relative quantity of protein was calculated after normalization to β-actin. Gp91^phox ^expression was markedly increased in the ischemic brain tissue, which was significantly inhibited by NBO. *P < 0.05 versus normoxic (NA) Non-I group, n = 6; ^#^p < 0.05 versus normoxic (NA) I group, n = 6.

**Figure 2:**
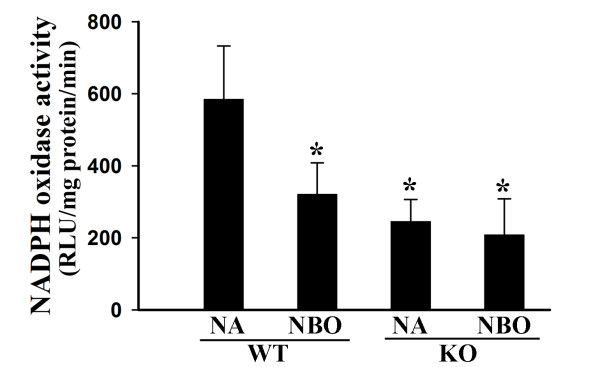
**NBO treatment reduces NADPH oxidase activity in ischemic hemispheric tissue of the wild-type mice (WT), but not gp91^phox ^knock-out mice (KO)**. Mice were subjected to 90-min MCAO with 22.5 hrs of reperfusion, NBO treatment or normoxia (NA) was given during 90-min MCAO. NADPH oxidase activity in ischemic hemispheric microvessels was assayed using lucigenin-enhanced chemiluminescence. NBO treatment significantly reduced NADPH oxidase activity in WT mice, but not in gp91^phox ^KO mice which already showed significantly low NADPH oxidase activity. *p < 0.05 versus NA-WT, n = 6.

Gp91^phox ^(or Nox2) containing NADPH oxidase is an important source of ROS in the brain [34]. We tested the effect of cerebral ischemia and reperfusion on the expression of this protein. As shown in Figure 1, gp91^phox ^protein level was significantly increased in the ischemic brain tissue in the wild-type mice after 90-min MCAO with 22.5-hr reperfusion. Interestingly, when mice were given NBO during the ischemic duration, this increase in gp91^phox ^was significantly reduced. Since gp91^phox ^is the catalytic unit of NADPH oxidase, we speculated that gp91^phox ^deletion or its inhibition by NBO would result in reduced NADPH oxidase activity. Indeed, as shown in Figure 2, gp91^phox ^knockout mice or NBO-treated wild-type mice showed significant reduction in the enzymatic activities of NADPH oxidase in the ischemic brain tissue. Importantly, NBO treatment did not induce further reduction in NADPH oxidase activity in gp91^phox ^knockout mice. These results indicate that NBO inhibits Nox2 containing NADPH oxidase in the ischemic brain.

### NBO protects the BBB against ischemic damage via acting on gp91^phox^

**Figure 3:**
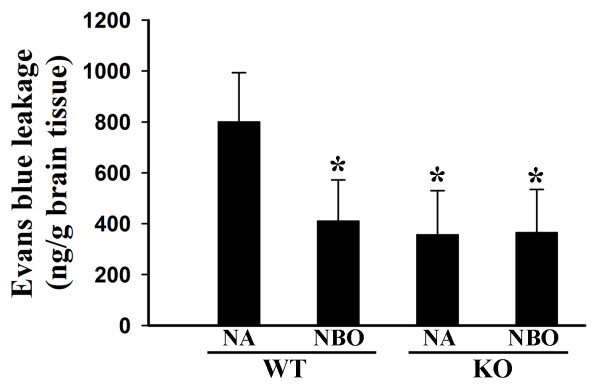
**NBO treatment or gp91^phox ^knock-out significantly reduces BBB disruption after 90-min MCAO with 22.5 hrs of reperfusion**. Evans blue leakage in the brain tissue was quantified according to the external standard curve which was generated by plotting the fluorescence intensity against the concentrations of Evans blue. Evans blue leakage was expressed as per gram of brain tissue (ng/g). Compared to the normoxic wild-type group (NA-WT), NBO-treated or gp91^phox ^knock-out (KO) mice showed a significant less amount of Evans blue extravasation. However, NBO did not further reduce Evans blue leakage in gp91^phox ^KO mice. *p < 0.05 versus NA WT group, n = 7.

Gp91^phox ^containing NADPH oxidase has been shown to contribute to BBB damage in animal stroke models. We speculated that inhibition of gp91^phox ^containing NADPH oxidase by NBO could resulted in reduction in ischemic BBB injury. We quantitated the extent of BBB damage by measuring the difference of Evans blue content between the ischemic and nonischemic hemispheres. As expected, 90-min MCAO with 22.5 hr-reperfusion induced significant amounts of Evans blue leakage in the wild-type mice, and much reduced leakage was observed in gp91^phox ^knockout mice (Figure 3). Interestingly, NBO treatment during MCAO significantly reduced Evans blue leakage in wild-type mice, but not in gp91^phox ^knockout mice MCAO (Figure 3). These results indicate that NBO-afforded BBB protection depends on its inhibition of gp91^phox ^containing NADPH oxidase.

### Gp91^phox ^containing NADPH oxidase contributes to MMP-9 induction in focal cerebral ischemia

**Figure 4:**
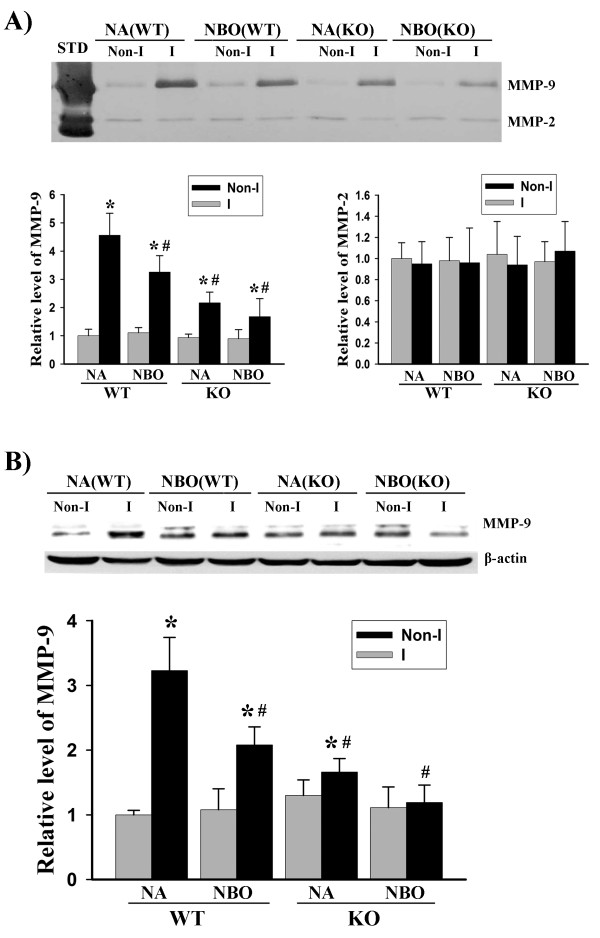
**NBO treatment or gp91^phox ^knock-out significantly reduces MMP-9 induction in ischemic brain tissue after 90-min MCAO with 22.5 hrs of reperfusion**. **A) **Hemispheric brain tissue was homogenized for analyzing MMP-2 and 9 levels with gel gelatin zymography. Upper panel: representative gelatin zymograms showing the expression of proforms of MMP-2 and 9 in Nonischemic (Non-I) and ischemic (I) brain tissues in normoxic wild-type (NA-WT), NBO-treated WT (NBO-WT), normoxic gp91^phox ^knock-out (NA-KO) and NBO-KO mice. STD is a mixture of human standard MMP-2 and 9. Bottom panels: the band intensities of MMP-2 and 9 were quantified. MMP-9 was significantly induced in the ischemic brain of NA-WT mice, and this induction was significantly inhibited by NBO or gp91^phox ^KO. The combination of NBO and gp91^phox ^KO led to a further, but not significant, reduction in MMP-9 compared each modulation alone (Left bottom panel). No significant changes were observed in MMP-2 for all groups (Right bottom panel). *p < 0.05 versus Non-I, n = 6; ^#^p < 0.05 versus NA-WT, n = 6. **B) **Hemispheric brain tissue was homogenized for analyzing MMP-9 protein level with western blot. Upper panel: representative blots of MMP-9 protein in hemispheric brain tissue obtained from NA-WT, NBO-WT, NA-KO and NBO-KO mice. β-actin served as a protein loading control. Bottom panel: the relative quantity of MMP-9 protein was calculated after normalization to β-actin. *p < 0.05 versus Non-I, n = 6; ^#^p < 0.05 versus NA-WT, n = 6.

To further understand the role of gp91^phox ^containing NADPH oxidase in ischemic BBB injury, we attempted to determine its involvement in MMP-9 induction in ischemic brain tissue using gp91^phox ^knockout mice. MMP-9 is a well-recognized molecule implicated in the proteolytic disruption of the BBB in ischemic stroke [35]. Gel zymography (Figure 4A) and western blot (Figure 4B) showed that cerebral ischemia and reperfusion induced remarkable increases in MMP-9 activity and its protein levels in the ischemic brain tissue in wild-type mice, while this MMP-9 induction was significantly, but not completely, inhibited when gp91^phox ^was knocked out. Different from its effect on BBB damage, NBO treatment not only inhibited MMP-9 induction in wild-type mice, but also caused further reduction, though not significant, in MMP-9 in gp91^phox ^knockout mice. Interestingly, at this relative late reperfusion time points (24 hrs after stroke onset), MMP-2 appeared to be constitutively expressed at much lower levels compared with MMP-9, and was not affected by ischemia and reperfusion (Figure 4A). These results suggest that MMP-9 induction is partially mediated by gp91^phox ^containing NADPH oxidase, and besides NADPH oxidase, NBO might inhibit MMP-9 induction through other unknown mechanisms.

### Gp91^phox ^containing NADPH oxidase contributes to occludin, but not claudin-5 degradation in focal cerebral ischemia

**Figure 5:**
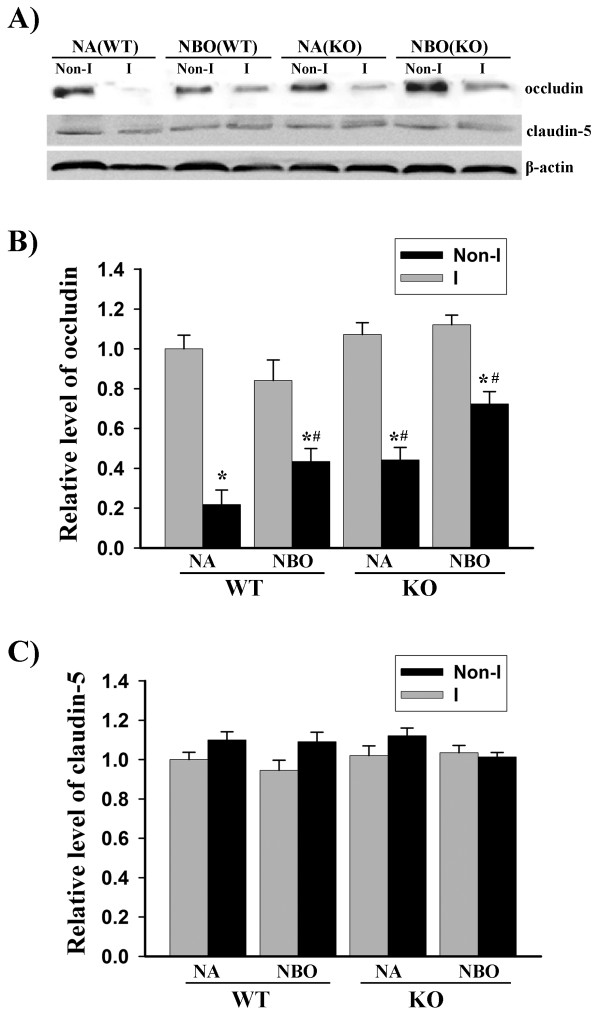
**NBO treatment or gp91^phox ^knock-out significantly reduces occludin degradation in ischemic brain tissue after 90-min MCAO with 22.5 hrs of reperfusion**. Hemispheric brain tissue was homogenized for analyzing tight junction proteins occludin and claudin-5 with western blot. **A) **Representative blots of occludin, claudin-5 and the corresponding β-actin showing their changes in Nonischemic (Non-I) and ischemic (I) brain tissues in normoxic wild-type (NA-WT), NBO-treated WT (NBO-WT), normoxic gp91^phox ^knock-out (NA-KO) and NBO-KO mice. **B) **The relative quantity of occludin protein was calculated after normalization to β-actin. Occludin protein level was significantly reduced in the ischemic brain of NA-WT mice, and this reduction was significantly reversed by NBO or gp91^phox ^KO. *p < 0.05 versus Non-I, and ^#^p < 0.05 versus NA-WT, n = 6. **C) **No significant changes were observed in claudin-5 protein for all groups.

Transmembrane protein occludin and claudins are the key molecules forming the seal between adjacent endothelial cells of the BBB. Using gp91^phox ^knockout mice, we tested the effect of gp91^phox ^containing NADPH oxidase on tight junction protein occludin and claudin-5. Consistent with our results obtained from ischemic stroke rats [4], ischemia and reperfusion induced a reduction in occludin protein, but not claudin-5, in wild-type mice, and NBO treatment significantly reversed this reduction (Figure 5). Considering the fact that occludin is substrate of MMP-9 and reduced MMP-9 induction in the ischemic brain of gp91^phox ^knockout mice (Figure 4), we speculated that gp91^phox ^knockout could lead to a reduction in occludin loss in ischemic brain tissue. Indeed, 90-min MCAO with 22.5 hrs of reperfusion induced occludin degradation to much less degree in gp91^phox ^knockout mice than normoxic wild-type mice (Figure 5). Similar to their effect on MMP-9 induction (Figure 4), the combination of NBO and gp91^phox ^knockout led to a further, but not significant, reduction in occludin protein loss in the ischemic tissue compared to each manipulation alone (Figure 5). No significant effects were observed for NBO or gp91^phox ^knockout on claudin-5 protein. These results suggest that gp91^phox ^containing NADPH oxidase is implicated in occludin degradation in the ischemic brain.

## Discussion

Using gp91^phox ^knockout mice, the present study unambiguously demonstrates that Nox2-containing NADPH oxidase is implicated in MMP-9 induction, occludin degradation and BBB disruption in focal cerebral ischemia and reperfusion. Moreover, Inhibition of Nox2 appears to be fully responsible for the protective effects of NBO treatment on the BBB, while it only partially accounts for NBO inhibitory effects on MMP-9 induction.

Increased ROS generation is a common event in cerebral ischemia contributing to ischemic brain injury [36-38]. Accumulating evidence has shown that NADPH oxidase is an important source of ROS in the brain, and inhibition of this enzyme can effectively reduce ischemic damage to neuronal tissue and the BBB [16,22-25,27,32]. Several NADPH oxidase homologs including Nox1, Nox2 and Nox4 have been found in the brain, and their roles in ischemic brain damage are quite different. In a mouse MCAO model, Nox1 knockout was found to have no effect on neurological deficit, total or subcortical cerebral infarct volume or edema volume, while contributed to the development of a 4-fold greater cortical infarct volume [39]. Nox4 may play an important role in ischemia/reperfusion-induced neoangiogenesis during stroke recovery [22]. On the contrary, Nox2-containing NADPH oxidase is shown to critically contribute to ischemic neuronal tissue injury and BBB disruption [16,18,27,32]. In the present study, we found that gp91^phox ^(or Nox2) expression was significantly up-regulated in ischemic mouse brain, and knockout of Nox2 resulted in a significant reduction in NADPH oxidase activity in ischemic brain tissue. These data indicate that Nox2 containing NADPH oxidase is activated during cerebral ischemia and reperfusion in mice.

The mechanisms by which NADPH oxidase contribute to ischemic brain damage, particularly its damage to the BBB, are currently under active investigation. As an important source of ROS in the brain, NADPH oxidase is readily involved in ischemic BBB damage through enhancing oxidative damage to the lipid bilayer membrane of the neurovascular cells. In addition, NADPH oxidase-derived ROS can also act as stimulators and activators to MMPs, thus enhancing their proteolytic degradation to the BBB [40]. MMPs are well-recognized effector molecules implicated in BBB damage during ischemic stroke [31,41]. Among MMP family members, gelatinases MMP-2 and 9 have been a research focus in ischemic stroke because of their substrate specificity for fibronectin, laminin, collagen type IV and tight junction proteins, which are structural components of the BBB [42,43]. Our results that gp91^phox ^knockout mouse exhibited a significant reduction in MMP-9 induction in the ischemic brain suggest an important role of Nox2 containing NADPH oxidase in MMP-9 upregulation in focal cerebral ischemia. We found that, unlike MMP-9, MMP-2 was constantly expressed at low levels in the brain tissue of both wild-type and gp91^phox ^knockout mice, and its expression was not affected by cerebral ischemia and reperfusion or gp91^phox ^knockout. These results demonstrate that at 24 hrs after stroke onset, MMP-9, rather than MMP-2, is the major gelatinase produced in the ischemic brain. Consistent with our findings, an early study showed that MMP-2 was the major gelatinase contributing to early BBB disruption, while MMP-9 appeared to play an important role at late reperfusion time points [33].

Proteolytic disruption of the tight junction proteins by MMPs has been well documented in ischemic stroke [33,43-45]. Claudins and occludin are integral transmembrane tight junction proteins forming the seal between adjacent endothelial cells [46,47]. Our results indicate that occludin protein is greatly decreased in the ischemic brain tissue of the wild-type mice, while no change is observed for caludin-5. This is consistent with our previous results obtained from a rat stroke model [44]. In vitro incubation of tight junction proteins with purified MMP-2 and/or 9 has provided solid evidence that occludin is a substrate of MMP-2/9 [4,48]. However, the effects of MMP-2/9 on claudin-5 appear to be more complicated because controversial results have been reported [4,48-50]. In agreement with its inhibitory effects on MMP-9 induction, gp91^phox ^knockout leads to significant reduction in occludin protein level in ischemic brain tissue. In contrast, gp91^phox ^knockout has no effect on claudin-5 expression in both normal and ischemic conditions. These results suggest that Nox2 containing NADPH oxidase contributes to occludin degradation, and this effect is probably secondary to NADPH oxidase's action on MMP-9.

Normobaric oxygen has been shown to be very effective in reducing tissue infarction and BBB damage in focal cerebral ischemia [1-4,8,9,11,51]. Increased oxidative stress has been an important concern with oxygen therapy. Interestingly, recently studies indicate that NBO treatment does not increase ROS production [2,3,51], and it may even decrease ROS generation in the ischemic penumbra [3]. In a recently study on ischemic stroke rats, we found that NBO treatment inhibited gp91^phox ^(or Nox2) expression and MMP-9 induction and reduced BBB disruption. To definitively clarify whether NBO acts on Nox2 to exert its BBB protection, we tested NBO's effects when gp91^phox ^was genetically deleted. Our results demonstrate that NBO significantly reduced gp91^phox ^protein expression and NADPH oxidase activity in the ischemic tissue; however, it did not cause any further reductions in BBB damage and NADPH oxidase activity when gp91^phox ^was knocked out. Since MMP-9 induction and occludin degradation in the ischemic brain was closely linked to Nox2, NBO's effects on these molecules were also diminished in gp91^phox ^knockout mice. These results suggest that NBO indeed acts on Nox2 containing oxidase to protect the BBB against ischemic damage. Under ischemic conditions, several mechanisms have been proposed to activate Nox2, such as phosphatidylinositol-3-kinase/AKT-dependent NF- κB and HIF-1 α pathways [52], inflammatory cytokine IL-1β [17] and metabotropic glutamate receptor 1 [53]. Our previous findings that NBO treatment improved ischemic tissue oxygenation by maintaining penumbral pO_2 _level close to the preischemic level [3] suggest that NBO may inhibit Nox2 activation through suppressing the above pathways secondary to NBO's effects on improving tissue oxygenation. Future studies are required to test these possibilities.

In conclusion, our results demonstrate that Nox2 containing NADPH oxidase critically contributes to ischemic BBB damage, and inhibiting Nox2 is an important mechanism underlying NBO-afforded BBB protection in transient cerebral ischemia and reperfusion.

## Competing interests

The authors declare that they have no competing interests.

## Authors' contributions

WL participated in the design of the study, obtained partial funding, and written up the manuscript. QC carried out all the animal experiments. JL participated in data processing and manuscript writing. KJL participated in the overall design of the study and manuscript writing, and obtained partial funding. All authors have read and approved the final manuscript.
